# Targeting VEGFR1- and VEGFR2-expressing non-tumor cells is essential for esophageal cancer therapy

**DOI:** 10.18632/oncotarget.2781

**Published:** 2014-12-17

**Authors:** Wen Wen Xu, Bin Li, Alfred KY Lam, Sai Wah Tsao, Simon YK Law, Kwok Wah Chan, Qiu Ju Yuan, Annie LM Cheung

**Affiliations:** ^1^ Department of Anatomy, Li Ka Shing Faculty of Medicine, The University of Hong Kong, Pokfulam, Hong Kong SAR, China; ^2^ The University of Hong Kong-Shenzhen Institute of Research and Innovation (HKU-SIRI), China; ^3^ Centre for Cancer Research, Li Ka Shing Faculty of Medicine, The University of Hong Kong, Pokfulam, Hong Kong SAR, China; ^4^ Department of Pathology, Griffith Medical School and Griffith Health Institute, Gold Coast Campus, Gold Coast, QLD 4222, Australia; ^5^ Department of Surgery, Li Ka Shing Faculty of Medicine, The University of Hong Kong, Pokfulam, Hong Kong SAR, China; ^6^ Department of Pathology, Li Ka Shing Faculty of Medicine, The University of Hong Kong, Pokfulam, Hong Kong SAR, China

**Keywords:** Tumor angiogenesis, Bone marrow-derived cells, Tumor microenvironment, VEGF receptors, Antibody therapy

## Abstract

Increasing appreciation of tumor heterogeneity and the tumor-host interaction has stimulated interest in developing novel therapies that target both tumor cells and tumor microenvironment. Bone marrow derived cells (BMDCs) constitute important components of the tumor microenvironment. In this study, we aim to investigate the significance of VEGFR1- and VEGFR2-expressing non-tumor cells, including BMDCs, in esophageal cancer (EC) progression and in VEGFR1/VEGFR2-targeted therapies. Here we report that VEGFR1 or VEGFR2 blockade can significantly attenuate VEGF-induced Src and Erk signaling, as well as the proliferation and migration of VEGFR1^+^ and VEGFR2^+^ bone marrow cells and their pro-invasive effect on cancer cells. Importantly, our *in vivo* data show for the first time that systemic blockade of VEGFR1^+^ or VEGFR2^+^ non-tumor cells with neutralizing antibodies is sufficient to significantly suppress esophageal tumor growth, angiogenesis and metastasis in mice. Moreover, our tissue microarray study of human EC clinical specimens showed the clinicopathological significance of VEGFR1 and VEGFR2 in EC, which suggest that anti-VEGFR1/VEGFR2 therapies may be particularly beneficial for patients with aggressive EC. In conclusion, this study demonstrates the important contributions of VEGFR1^+^ and VEGFR2^+^ non-tumor cells in esophageal cancer progression, and substantiates the validity of these receptors as therapeutic targets for this deadly disease.

## INTRODUCTION

Angiogenesis is one of the important hallmarks of cancer [1]. It has been more than 40 years since Judah Folkman published his classic article “Tumor angiogenesis: therapeutic implications” [2], and it is now well recognized that vascular endothelial growth factor (VEGF), which exerts its functions mainly by binding to both VEGF receptor 1 (VEGFR1) and VEGF receptor 2 (VEGFR2), is the master regulator of the process. A number of studies including ours have established the clinical significance of VEGF in different types of human cancers [3, 4]. In recent years, there is also a growing appreciation of the close interaction between cancer cells and various elements in the tumor microenvironment, and the importance of bone marrow-derived cells (BMDCs) as components of the tumor microenvironment that contribute significantly to tumor vascularization [5, 6]. The formation of blood vessels involves two processes: angiogenesis which is the sprouting of new blood vessels from pre-existing ones, and vasculogenesis which is the recruitment of circulating endothelial progenitor cells (EPCs) to form new blood vessels [7]. Moreover, bone marrow-derived VEGFR1-positive (VEGFR1^+^) hematopoietic progenitor cells (HPCs), in response to tumor-secreted growth factors and cytokines, may be recruited to distant organs to form clusters and create a permissive microenvironment that is structurally and functionally conducive to cancer metastasis [8, 9]. In addition, the progression from micro- to macro-metastasis depends on the establishment of functional vasculature, which involves the mobilization and recruitment of bone marrow-derived VEGFR2-positive (VEGFR2^+^) EPCs to the metastatic site [10, 11]. However, the paracrine effects of VEGFR1^+^ and VEGFR2^+^ BMDCs on cancer cells is unknown, and their contribution to human cancer pathogenesis remains to be further elucidated.

Esophageal squamous cell carcinoma (ESCC) is one of the most aggressive malignancies in the world and angiogenesis is a major clinical feature of aggressive ESCC [12, 13]. ESCC carries a very poor prognosis because many cases go undetected until the disease is at an advanced stage. Recent evidence indicates that the expression of VEGFR1 and VEGFR2 is correlated with prognosis of patients in various types of cancers [14–16]. However, little is known about the expression patterns and clinical significance of VEGFR1 and VEGFR2 in ESCC. Whether blockade of VEGFR1 or VEGFR2 can inhibit progression of ESCC is still unknown.

Thus, in this study, we aim to investigate the significance of VEGFR1- and VEGFR2-expressing non-tumor cells including BMDCs, which constitute important components of the tumor microenvironment, in esophageal cancer progression, and to determine whether targeting these cells could suppress tumor angiogenesis and progression. The outcome of this study enhances our understanding of the functional significance of VEGFR1^+^/VEGFR2^+^ non-tumor and tumor cells, which may serve as therapeutic targets in ESCC.

## RESULTS

### Targeting host VEGFR1 and VEGFR2 can suppress growth of human esophageal tumor xenograft in mice

**Figure 1:**
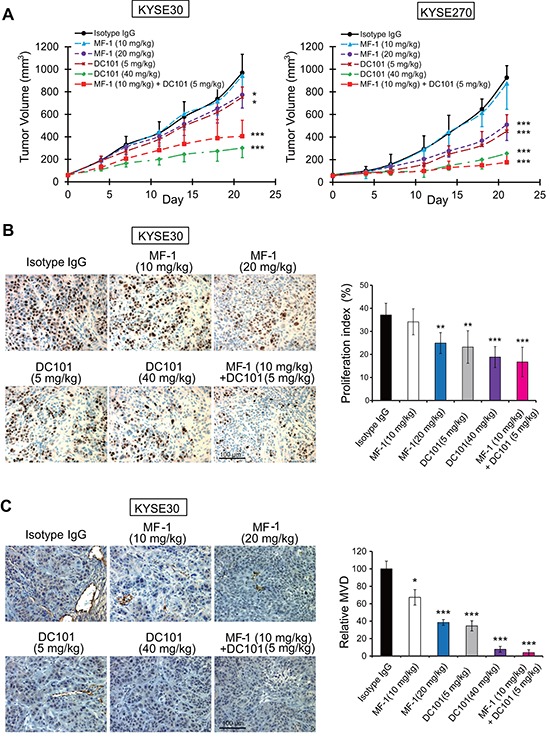
Blockade of VEGFR1 and VEGFR2 suppressed growth of human ESCC xenografts in nude mice **(A)** Growth curves of KYSE30 and KYSE270 tumor xenografts. **(B)** Immunohistochemical analysis of the Ki-67 proliferation index in KYSE30 xenografts. **(C)** CD31-positive cells demonstrating micro-vessel density (MVD) in KYSE30 xenografts. Bars, SD; *, *P* < 0.05; **, *P* < 0.01, ***, *P* < 0.001 compared with isotype IgG-treated mice.

To study the roles of VEGFR1- and VEGFR2-expressing non-tumor cells in the development of esophageal cancer, we made use of mouse-specific antibodies to determine if blockade of host VEGFR1 and VEGFR2 had any effects on growth of xenografted human ESCC tumors in mouse models. Nude mice bearing subcutaneous human ESCC tumor xenografts derived from KYSE30 and KYSE270 cells were treated with anti-mouse VEGFR1 and VEGFR2 neutralizing antibodies. The results showed that the antibodies MF-1 or DC101 significantly suppressed tumor growth in human ESCC tumour xenografts in a dose-dependent manner. When a combination of MF-1 and DC101 was applied at low doses (10 mg/kg and 5 mg/kg respectively), improved tumor response was obtained compared to treatment with either one of the antibodies (Figure 1A). We also found that MF-1 and DC101, alone or in combination, could significantly reduce Ki-67-positive tumor cells in the KYSE30 and KYSE270 xenografts, suggesting that blockade of host VEGFR1/VEGFR2 could inhibit tumor cell proliferation (Figure 1B and Supplementary Figure S1A). Since the mouse-specific MF-1 and DC101 antibodies exerted no significant inhibitory effect on proliferation of human ESCC cells *in vitro* (Supplementary Figure S2), the observed anti-tumor activity was unlikely to be a direct effect on human cancer cells but could be mediated by mouse non-tumor cells that expressed VEGFR1 or VEGFR2. To rule out the possibility of MF-1 and DC101 cross-reacting with human epidermal growth factor receptors (EGFR) expressed on cancer cells, the tumor xenografts were subjected to Western blot analysis of phosphorylation form of EGFR (p-EGFR) and EGFR. The results showed no significant difference in the expression of p-EGFR and EGFR between the treated and control groups (Supplementary Figure S3), thus confirming that the suppressive effects of MF-1 and DC101 antibodies on tumor growth were not due to blockade of EGFR on cancer cells. In addition, we found that MF-1 and DC101 significantly decreased micro-vessel density (MVD), as identified by CD31 (Figure 1C and Supplementary Figure S1B), which was indicative of repressed tumor angiogenesis. With the exception of the group treated with high dosage DC101, which showed a slight but statistically insignificant weight loss after two weeks, there was no obvious difference in body weight among the other groups (Supplementary Figure S4A). Histological evaluation of the vital organs of the mice, including lungs, liver and kidneys did not reveal overt changes in morphology (Supplementary Figure S4B), suggesting that the antibody treatments had no toxic effects.

### Inhibitory effects of VEGFR1 and VEGFR2 antibodies on tumor growth are partly attributed to anti-angiogenic influence

**Figure 2:**
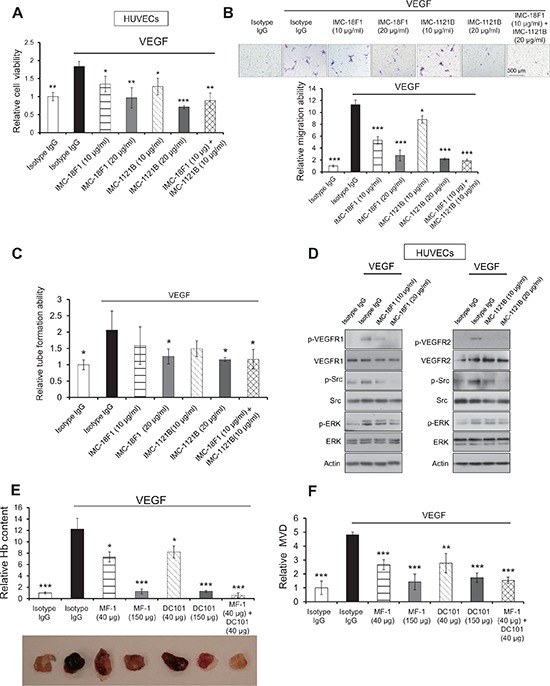
Blockade of VEGFR1 and VEGFR2 inhibited VEGF-induced angiogenesis *in vitro* and *in vivo* **(A–C)** The proliferation **(A)**, migration **(B)**, tube formation **(C)** of human umbilical vein endothelial cells (HUVECs) were evaluated after treatment with IMC-18F1, IMC-1121B alone or in combination, in the presence or absence of recombinant VEGF. **(D)** Lysates from HUVECs were collected for Western blot analysis of p-VEGFR1 (Tyr1213), p-VEGFR2 (Tyr1175), VEGFR1, VEGFR2, p-Erk (Thr202/Tyr204), p-Src (Tyr416), Erk and Src. **(E)** Matrigel plug assay was performed to assess the anti-angiogenic effect of MF-1 and DC101 *in vivo*. The hemoglobin (Hb) content in the matrigel plugs was measured and expressed relative to that of isotype control without VEGF treatment. **(F)** Determination of CD31 MVD in the excised matrigel plugs. Bars, SD; *, *P* < 0.05; **, *P* < 0.01, ***, *P* < 0.001 compared with the cells or mice treated with VEGF and isotype IgG.

Decrease in tumor MVD in the MF-1 or DC101-treated animals (Figure 1C and Supplementary Figure S1B) prompted us to further examine whether the anti-tumor effects were due to inhibition of angiogenesis. We found that treatment with antibodies directed against human VEGFR1 and VEGFR2 (i.e. IMC-18F1 and IMC-1121B, respectively) reduced the proliferation of VEGF-stimulated HUVECs in a dose-dependent manner. Notably, a combination of both antibodies at low doses produced inhibitory effect comparable to high dose single-antibody treatments (Figure 2A). Moreover, the data from migration chamber assay showed that IMC-18F1 or IMC-1121B used alone at higher doses, or in combination with each other at low doses, abolished VEGF-stimulated migration of HUVECs (Figure 2B). Our results also showed that blockade of VEGFR1 and/or VEGFR2 significantly and dose-dependently decreased tube formation ability of HUVECs under VEGF stimulation (Figure 2C and Supplementary Figure S5A). Western blot analysis showed that VEGF upregulated the expression of p-VEGFR1, p-VEGFR2, p-Src and p-ERK in HUVECs, and that VEGFR1 and VEGFR2 antibodies attenuated these effects (Figure 2D).

*In vivo* matrigel plug assay was performed to assess anti-angiogenic effect upon blockade of host VEGFR1 and VEGFR2. The results showed that MF-1 and/or DC101 inhibited VEGF-induced neovascularization in tumor cell-free matrigel plugs (i.e. in the absence of paracrine influence from tumor cells), as evidenced by the significantly lower hemoglobin content (Figure 2E) and decreased MVD (Figure 2F and Supplementary Figure S5B) in the matrigel plugs. These data confirm that the tumor-suppressive effects of MF-1 and DC101 were due, at least in part, to inhibition of angiogenesis.

### Targeting host VEGFR1 and VEGFR2 results in a reduction of VEGFR1^+^ hematopoietic progenitor cells and VEGFR2^+^ endothelial progenitor cells

**Figure 3:**
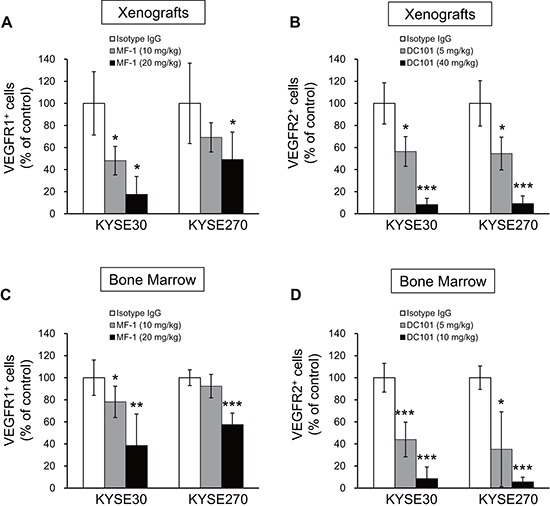
Flow cytometric analysis of percentage of VEGFR1^+^ and VEGFR2^+^ cells in tumor xenografts and bone marrow **(A–D)** Treatment with MF-1 and DC101 reduced the number of VEGFR1^+^ and VEGFR2^+^ cells, respectively, in tumor xenografts (A, B) and in bone marrow (C, D). Bars, SD; *, *P* < 0.05; **, *P* < 0.01, ***, *P* < 0.001 compared with isotype IgG-treated mice.

Although VEGFRs are normally found on vascular endothelial cells, bone marrow progenitor cells which express VEGFR1 and VEGFR2 have been reported to play an important role in the development of cancer. The success of MF-1/DC101 treatment in retarding tumor growth led us to explore whether bone marrow-derived VEGFR1^+^ HPCs and VEGFR2^+^ EPCs cells were involved in mediating these effects. Flow cytometric analysis of single-cell suspensions of tumor xenografts showed that treatment with MF-1 and DC101 led to dose-dependent decrease in the number of host VEGFR1^+^ and VEGFR2^+^ cells respectively (Figure 3A–3B). Notably, we also observed a dose-dependent decrease of VEGFR1^+^ and VEGFR2^+^ cells in the bone marrow of treated mice (Figure 3C–3D).

### VEGFR-immunoneutralization inhibits VEGF-stimulated proliferation and migration of VEGFR1^+^ and VEGFR2^+^ bone marrow-derived cells

**Figure 4:**
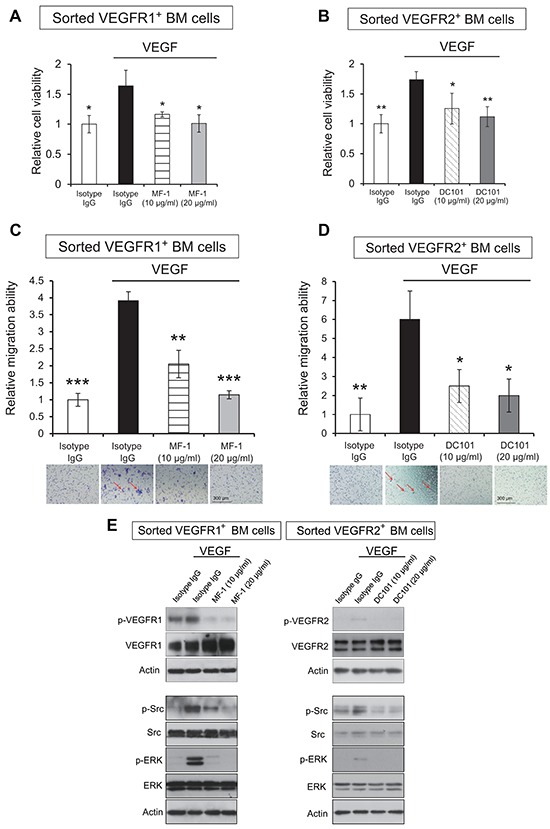
MF-1 and DC101 suppressed the proliferation and migration of sorted VEGFR1^+^ and VEGFR2^+^ bone marrow cells **(A, B)**, VEGF-stimulated proliferation of sorted VEGFR1^+^ and VEGFR2^+^ bone marrow cells was determined 9 days after treatment with two different concentrations of MF-1 or DC101. **(C, D)**, Migration chamber assay was performed to determine the inhibitory effect of MF-1 and DC101 on the migration ability of VEGFR1^+^ and VEGFR2^+^ bone marrow cells, respectively. The migrated cells were indicated by the arrows. **(E)** Blocking VEGFR1/VEGFR2 abolished VEGF-stimulated activation of Src and Erk in bone marrow cells. Sorted mouse VEGFR1^+^ and VEGFR2^+^ cells were collected for Western blot analysis of VEGFR1/VEGFR2, Erk, Src and their phosphorylated forms, including p-VEGFR1 (Tyr1213), p-VEGFR2 (Tyr1175), p-Erk (Thr202/Tyr204) and p-Src (Tyr416). Actin served as loading control. Bars, SD; *, *P* < 0.05; **, *P* < 0.01, ***, *P* < 0.001 compared with the cells treated with VEGF and isotype IgG.

It was reported that VEGFR1^+^ and VEGFR2^+^ BMDCs contribute to tumor angiogenesis and progression. Such function is likely dependent on increased proliferation and migration of these cells so as to facilitate their recruitment into developing tumors as well as metastatic sites during cancer progression. There is as yet no report on the direct effects of VEGFR blockade on BMDCs. We sorted VEGFR1^+^ and VEGFR2^+^ bone marrow cells from mouse bone marrow (Supplementary Figure S6) for *ex vivo* culture, and studied the effects of VEGF stimulation and VEGFR-immunoneutralization on cell proliferation and migration. The data showed that treatment with MF-1 and DC101 significantly attenuated VEGF-stimulated proliferation of VEGFR1^+^ and VEGFR2^+^ bone marrow cells, respectively (Figure 4A–4B). The results from migration chamber assay showed that the antibodies suppressed the chemotaxis of corresponding VEGFR1^+^ and VEGFR2^+^ bone marrow cells in response to VEGF (Figure 4C–4D). Western blot analysis showed that MF-1 and DC101 abolished VEGF-induced phosphorylation of VEGFR1 and VEGFR2, respectively, in sorted VEGFR1^+^ and VEGFR2^+^ bone marrow cells. In addition, our results showed that VEGF increased the expressions of p-Src and p-Erk in these cells, and that the effects were abrogated by blockade of VEGFR1 and VEGFR2 (Figure 4E), which were consistent with the effects on HUVECs (Figure 2D).

### Blockade of VEGFR1 and VEGFR2 in human umbilical vein endothelial cells and bone marrow-derived cells reduces their pro-invasive effect on cancer cells

**Figure 5:**
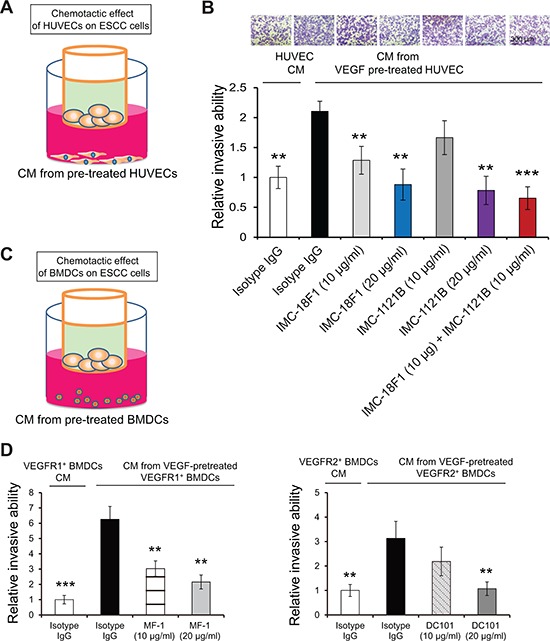
The pro-invasive effect of HUVECs or bone marrow cells on cancer cells **(A, B)** Invasion chamber assay was used to evaluate the invasion of KYSE270 cells attracted by HUVECs in the presence or absence of IMC-18F1 and IMC-1121B. **(C, D)** The ability of sorted VEGFR1^+^ and VEGFR2^+^ bone marrow cells to attract invading KYSE270 cancer cells was determined. Bars, SD; **, *P* < 0.01, ***, *P* < 0.001 compared with the cells treated with VEGF and isotype IgG.

Next we explored the significance of VEGFR1/VEGFR2 pathways in the interactions between cancer cells and endothelial cells as well as bone marrow cells in the tumor microenvironment. Invasion assay showed that the conditioned medium (CM) from VEGF-pretreated HUVECs could increase the invasive ability of ESCC cells and that the effect was abrogated in the presence of VEGFR1/VEGFR2 antibodies (Figure 5A–5B). In addition, to mimic the metastatic microenvironment in which BMDCs might exert paracrine influence on cancer cells, CM was collected from VEGF-stimulated VEGFR1^+^ and VEGFR2^+^ bone marrow cells for invasion chamber assay (Figure 5C). The results showed enhanced invasion of human ESCC cells within 24 h, and that blocking VEGFR1 on bone marrow cells with MF-1, or VEGFR2 with DC101, partially abolished this effect (Figure 5D). These data collectively indicate that VEGFR1^+^/VEGFR2^+^ endothelial and BMDCs play a vital role in the chemo-attraction of circulating cancer cells, and that blockade of VEGFR1/VEGFR2 could significantly prohibit this process.

### Blockade of host VEGFR1 and VEGFR2 reduces cancer metastasis

**Figure 6:**
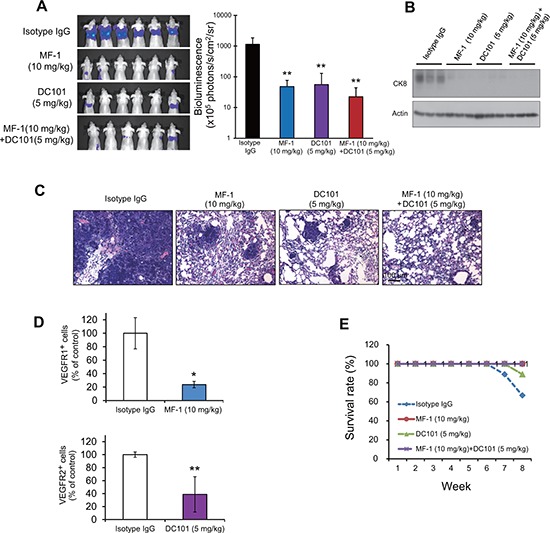
MF-1 and DC101 suppressed metastasis of ESCC cells **(A)** Metastasis of ESCC cells was quantified by bioluminescence imaging at the 8^th^ week after injection of KYSE150-Luc cells. **(B)** Western blot analysis of human-specific cytokeratin 8 protein in the lung extracts of 3 representative mice in each group. **(C)** Histological evaluation of lung metastasis (hematoxylin and eosin staining). **(D)** Flow cytometric analysis showed that MF-1 and DC101 significantly reduced the percentages of VEGFR1^+^ and VEGFR2^+^ cells, respectively, in the lungs. **(E)** The survival rates of mice over an 8-week period after introduction of cancer cells. Bars, SD; *, *P* < 0.05; **, *P* < 0.01, compared with isotype IgG-treated mice.

In light of the pro-invasive effect of HUVECs and BMDCs on cancer cells, we next determined whether blockade of host VEGFR1 and VEGFR2 could inhibit cancer metastasis. Using bioluminescence imaging in an experimental metastasis model, we found that treatment with MF-1 and/or DC101 significantly decreased the colonization of intravenously injected ESCC cells to the lungs, compared with control animals receiving isotype IgG (Figure 6A). This finding was confirmed by the reduction in expression of human-specific cytokeratin 8 protein which identified the human cancer cells (Figure 6B), and in the extent of macro-metastases in the lungs of the treatment groups (Figure 6C). In addition, we found that the MF-1 and DC101-treated groups had significantly lower percentage of VEGFR1^+^ and VEGFR2^+^ cells respectively in the lungs (Figure 6D). Furthermore, treatment with the antibodies was found to improve the survival rates of the mice (Figure 6E). Taken together, these data demonstrate that host VEGFR1^+^ and VEGFR2^+^ cells play an important role in metastasis, and that targeting these cells using neutralization antibodies can decrease distant metastases in ESCC.

### Clinical significance of VEGFR1 and VEGFR2 in ESCC

**Figure 7:**
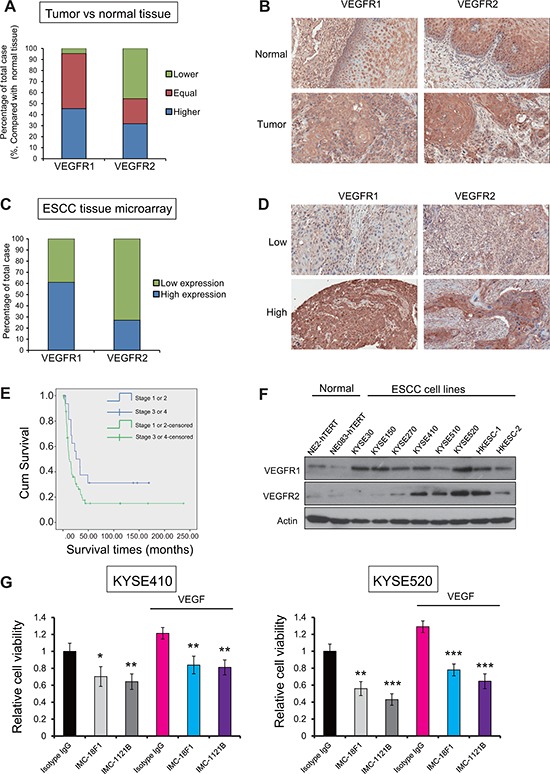
Clinical significance of VEGFR1 and VEGFR2 in ESCC **(A)** Comparison of VEGFR1 and VEGFR2 expression levels between 20 cases of ESCC and normal esophageal epithelium. **(B)** Representative histological features of case-matched ESCC and non-cancer esophageal epithelium. **(C)** Graph showing percentage of cases with low and high expressions of VEGFR1 and VEGFR2 in a TMA of 86 ESCC samples. **(D)** Histological features of ESCC with high or low expression of VEGFR1 and VEGFR2. **(E)** Kaplan-Meier analysis showed that the overall survival rates of patients with ESCC depend on pathological staging of ESCC patients. (*P* = 0.031). Patients with lower pathological stages (stage I or stage II) had longer survival time (survival time approximately 40 months when cumulative survival = 0.5) than patients with advanced pathological stages (stage III or stage IV) (survival time approximately 16 months when cumulative survival = 0.5). **(F)** The expression of VEGFR1 and VEGFR2 in a panel of ESCC cells lines and immortalized non-tumor esophageal epithelial cells. **(G)** The proliferation of two ESCC cell lines after IMC-18F1 and IMC-1121B treatments, with or without VEGF stimulation, was compared with that of isotype IgG-treated cells. Bars, SD; *, *P* < 0.05; **, *P* < 0.01, ***, *P* < 0.001.

Although our results showed that targeting host VEGFR1^+^ and VEGFR2^+^ non-tumor cells was sufficient to inhibit tumor growth, the notion that a therapy may be more effective if it can target both the cancer cells and its microenvironment prompted us to investigate the expression patterns and clinical significance of VEGFR1 and VEGFR2 in ESCC. Two of the 22 cases in the first TMA were excluded because the cancer-adjacent tissue sections did not contain esophageal epithelium. Of the remaining 20 cases of ESCC, 45% (9/20) and 35% (7/20) expressed higher VEGFR1 and VEGFR2, respectively, in the cytoplasm of cancer cells compared with cancer-adjacent normal esophageal epithelium (Figure 7A–7B).

To evaluate the clinicopathological impact and prognostic significance of VEGFR1 and VEGFR2 expressions, another tissue array containing 86 cases of ESCC collected prospectively by our group was used. High expression of VEGFR1 and VEGFR2 was found in 59% (51/86) and 28% (24/86) cases, respectively (Figure 7C–7D). Overall, 45/86 (52%) of the cases shared the same level of expression for both receptors, i.e. high expression of both VEGFR1 and high VEGFR2 (17 cases), or low expression of both VEGFR1 and VEGFR2 (28 cases). Furthermore, VEGFR1 expression was noted to be significantly correlated with the TMN pathological staging whereas VEGFR2 expression was associated with the presence of metastases (M stage) of the cancer (Table 1). The survival of the patients with ESCC was found to be associated with the pathological stages (*P* = 0.031) (Figure 7E). Other than these, there was no significant association with other clinical and pathological parameters including age, gender of the patients, tumor site, and tumor grade (Table 1).

**Table 1:** Correlation between VEGFR1 or VEGFR2 expressions and clinicopathological characteristics of 86 cases of ESCC

Parameters	VEGFR1				VEGFR2			
	Low	High	Total	*P* value	Low	High	Total	*P* value
*Number*	35	51	86	-	62	24	86	-
*Mean age*	61	65	-	0.067	62	64	-	0.32
*Mean diameter*	5.4	5.2	-	0.643	5.5	4.9	-	0.27
*Gender*								
Male	29	45	74		51	23	74	
Female	6	6	12	0.577	11	1	12	0.166
*Differentiation*								
Well and moderate	30	41	71		50	21	71	
Poor	5	10	15	0.577	12	3	15	0.542
*Location*								
Upper or middle	25	29	54		40	14	54	
Lower	10	22	32	0.183	22	10	32	0.626
*T-Stage*								
1/2	3	1	4		48	17	65	
3/4	32	50	82	0.3	14	7	21	0.58
*N-Stage*								
N0	11	11	22		18	4	22	
N1	24	40	64	0.325	44	20	64	0.283
*M-Stage*								
M0	34	50	84		62	22	84	
M1	1	1	2	1	0	2	2	**0.022**
*Stage*								
1	1	1	2		1	1	2	
2	10	6	16		14	2	16	
3	23	43	66		47	19	66	
4	1	1	2	**0.049**	0	2	2	0.051

Abbreviations: T, tumor; N, nodal; M, metastasis

### Roles of VEGFR1 and VEGFR2 expressions in cancer cell lines

We also compared a panel of ESCC cell lines and non-cancer esophageal epithelial cell lines for VEGFR1 and VEGFR2 expressions, and found that the majority of cancer cell lines expressed higher VEGFR1 and VEGFR2 compared with non-cancer esophageal epithelial cells (Figure 7F). Two ESCC cell lines with relative high expression of VEGFR1 and VEGFR2 were treated with VEGFR1 and VEGFR2 antibodies, and the results showed that proliferation of the treated cancer cells was significantly inhibited by the antibody treatments (Figure 7G), which suggests that ESCC cells are also valid targets for VEGFR1/VEGFR2 antibody therapies.

## DISCUSSION

In recent years, the role of BMDCs in development of cancer and their dialogue with cancer cells are under intensive investigation. Controversy exists as to whether BMDCs in the tumor microenvironment possess tumor-promoting or anti-tumor activities [17–19]. This may be partly explained by the complexity of their interaction with tumor cells. Although there are a number of reports documenting the recruitment of VEGFR1^+^ HPCs and VEGFR2^+^ EPCs to tumor and metastatic sites which facilitates cancer progression in murine models [11, 20], direct evidence of VEGFR1^+^ and VEGFR2^+^ BMDCs having paracrine effects on cancer cells is still lacking and the molecular pathways underlying their functions remain undefined. In this study, we showed for the first time that VEGF-induced bone marrow cells could instigate the mobility of cancer cells.

Although the activation of Src and Erk pathways are crucial players in oncogenesis through mediating multiple signaling pathways [21, 22], most studies investigated their functions in cancer cells. Little is known about their roles in bone marrow cells. In this study, we found that VEGF could activate the Src and Erk pathways in VEGFR1^+^ and VEGFR2^+^ BMDCs, and that blockade using corresponding VEGFR antibodies not only attenuated this effect, but also abolished the VEGF-induced proliferation and migration of sorted VEGFR1^+^ and VEGFR2^+^ BMDCs. The results suggest that activation of the Src and Erk pathways may contribute to the functions of VEGFR1^+^ and VEGFR2^+^ BMDCs in promoting cancer progression.

Cancer cells have long been regarded as the exclusive target in cancer therapy, but recent studies suggest that novel approaches that disrupt the tumor-host interactions may complement standard treatments to improve treatment efficacy [23]. A better understanding of the interactions between cancer cells and their local and systemic environments is therefore essential. Angiogenesis is the most obvious evidence of the tumor stroma playing a supportive role in tumor development, and targeting tumor angiogenesis has emerged as a promising anti-tumor strategy [24–26]. Since the introduction of the first effective therapeutic antibodies in cancer treatment (i.e. rituximab and trastuzumab), there has been an explosion of therapeutic antibodies. Till now, around 13 antibodies have been approved by the FDA as cancer drugs and many more are currently being evaluated in clinical trials [27]. These antibodies are currently used in the treatment of metastatic cancer such as colorectal cancer and breast cancer with good therapeutic responses. Compared with the small-molecule agents, antibody therapy generally has advantages such as longer duration of action and less inter-patient variability at a given dose from the perspective of pharmacokinetic studies [28]. In this study, we showed that blockade of host VEGFR1 or VEGFR2 by using the species-specific neutralizing antibodies could suppress ESCC tumor growth, angiogenesis and metastasis in preclinical models, thus providing strong evidence of the importance of tumor micro- and systemic environments in cancer progression.

It is widely accepted that VEGFR2 mediates most of the cellular responses to VEGF [29], while the downstream signaling of VEGFR1 is less well defined. Our results showed that simultaneous blockade VEGFR1 and VEGFR2 had co-adjuvant inhibitory effect on tumor growth and metastasis. This concurs with the notion that, due to the complexity of angiogenesis, multiple-targeting may be beneficial in the treatment of tumor angiogenesis and metastasis [30]. The remarkable anticancer effects and low toxicity of monoclonal antibodies in our preclinical models suggest that VEGFR1 and VEGFR2 antibodies could have a clinical role in treatment of ESCC.

Information on the clinicopathological significance of VEGFR1 and VEGFR2 in ESCC is scarce. Compared to earlier immunohistochemical studies of VEGFR1 and VEGFR2 expressions in human ESCC [31, 32], ours was conducted on the largest patient cohort. Our data showed that the expressions of both receptors were correlated with the pathological stages of the ESCC. Thus, targeting these receptors in metastasizing ESCC may be useful in the treatment of the disease. It is noteworthy that approximately one fifth of the ESCC showed high expression of both VEGFR1 and VEGFR2, which further emphasizes the merit of targeting both VEGFR1 and VEGRF2 in the treatment of ESCC. Our data collectively demonstrated for the first time the clinicopathological significance of VEGFR1 and VEGFR2 in ESCC and their potential to be druggable targets for this deadly disease. More importantly, the inhibitory effect of anti-human VEGFR antibodies on the human cancer cell proliferation (Figure 7G) corroborate that better tumor response may be obtained using therapeutic agents which are capable of targeting both the cancer microenvironment and the cancer cells. IMC-1121B (also known as Ramucirumab) was recently approved by FDA in April 2014 as second-line therapy for patients with gastric cancer.

In conclusion, our study demonstrated that VEGFR1^+^ HPCs and VEGFR2^+^ EPCs play significant functional roles in tumor progression, and that targeting the host non-tumor VEGFR- expressing cells could inhibit tumor growth, angiogenesis and metastasis. The cellular and molecular mechanisms of how cancer cells instigate the recruitment and mobilization of VEGFR1^+^ and VEGFR2^+^ BMDCs to developing tumors and to metastatic sites warrant further study.

## MATERIALS AND METHODS

### Cell lines

The cell lines recruited in this study included human ESCC cells, endothelial cells and immortalized esophageal epithelial cells. Human ESCC cell lines KYSE30, KYSE150, KYSE270, KYSE410, KYSE510, KYSE520 (DSMZ, Braunschweig, Germany) [33], as well as HKESC-1 [34] and HKESC-2 [35], were maintained in RPMI 1640 (Sigma, St Louis, MO) supplemented with 10% fetal bovine serum (Invitrogen, Gaithersburg, MD). Human umbilical vein endothelial cells (HUVECs) obtained from Invitrogen were cultured in M200/LSGS medium (Invitrogen). Human immortalized normal esophageal epithelial cell lines NE2-hTERT [36] and NE083-hTERT [37] were maintained in defined K-SFM (Invitrogen).

### Antibodies for immunoneutralization

Species-specific, non-cross-reactive monoclonal antibodies targeting mouse VEGFR1 (MF-1) and mouse VEGFR2 (DC101), as well as fully human monoclonal antibodies directed against human VEGFR1 (IMC-18F1) and human VEGFR2 (IMC-1121B), were provided by ImClone Systems (New York, NY) [38–41].

### *In vivo* tumorigenesis experiments

*In vivo* tumorgenesis and metastasis experiments were described previously [42, 43]. All animal care and experimental procedures were approved by the Committee on the Use of Live Animals in Teaching and Research, University of Hong Kong. Briefly, 1 × 10^6^ KYSE30 or 5 × 10^5^ KYSE270 cells suspended in a 1:1 mixture of phosphate buffered saline/Matrigel were subcutaneously injected into the flank of nude mice. When the tumors reached ~5 mm diameter, the mice were randomized into different groups then treated with indicated doses of neutralizing antibodies specific for mouse VEGFR1 (MF1) and VEGFR2 (DC101) intraperitoneally thrice weekly (*n* = 6 per group). During the experiment, the tumor volume and body weight were measured every 3 days. Tumor volumes were calculated with the equation V = (length × width^2^)/2. At the end of the experiments, the tumors, lungs, liver, and kidneys of the animals were collected for further analysis. Immunohistochemical analysis of proliferative index and MVD was performed using antibodies against Ki-67 (Dako, Mississauga, ON, Canada) and CD31 (Santa Cruz Biotechnology, Santa Cruz, CA), respectively.

### *In vivo* experimental metastasis experiments

In brief, 1 × 10^6^ luciferase-expressing KYSE150 cells (KYSE150-Luc) were injected intravenously into the systemic circulation of nude mice via the tail vein (*n* = 9 per group). After 7 days, the mice were treated with MF1 and/or DC101 every 3 days. Metastasis was monitored by bioluminescent imaging.

### *In vivo* matrigel plug assay

Matrigel plug assay was performed according to the method described by Passaniti *et al* [44]. See supplementary materials and methods for details.

### Preparation of cell suspensions from mouse bone marrow, tumor xenografts and lungs

See supplementary materials and methods for details.

### Fluorescence-activated cell sorting and analysis

Bone marrow cells, as well as cell suspensions of lungs and tumor xenografts were labeled with phycoerythrin-conjugated anti-mouse VEGFR1 (#141522, R&D Systems, Minneapolis, MN), anti-mouse VEGFR2 (#Avas12a1, eBioscience Inc., CA) or with corresponding isotype control antibody. See supplementary materials and methods for details.

### Western blot analysis and cell proliferation, migration, invasion assays

Western blot analysis, cell proliferation, migration and invasion assays were performed as previously described [45, 46]. Please see the supplementary materials and methods for details.

### Endothelial tube formation assay

The tube formation assay was performed according to Bae et al. [47]. Please see the supplementary materials and methods for details.

### Tissue microarray analysis

A human esophageal cancer tissue microarray (TMA) containing 22 cases of ESCC with cancer-adjacent normal esophageal tissue (ES722, Biomax, Rockville, MD), as well as another TMA containing 86 cases of primary ESCC were used to investigate the expression of VEGFR1 and VEGFR2. The latter TMA was constructed from ESCC of 86 patients who had undergone esophagectomy between 1991 and 1998 at the Department of Surgery, University of Hong Kong Medical Centre, Queen Mary Hospital, Hong Kong. None of these patients had received neo-adjuvant chemotherapy or radiotherapy. Immunohistochemical approach was taken to detect the expression of VEGFR1 and VEGFR2 using anti-VEGFR1 (#AF321, R&D Systems) and anti-VEGFR2 (#2479, Cell Signaling Technology, Beverly, MA) antibodies respectively. The immunohistochemical staining for VEGFR1 and VEGFR2 expressions was classified into four categories: no or negligible staining (score 0), weakly positive with less than 10% tumor cells (or esophageal epithelial cells for sections of cancer-adjacent tissue) stained (score +), moderate intensity with 10 to 30% cells stained (score ++), and strong staining intensity in > 50% cells (score +++). Cases with scores of 0, + or ++ were grouped as “low” expression, whereas those with a score of +++ were regarded as having high expression.

### Statistical analysis

All *in vitro* experiments and assays were repeated at least 3 times, and the data were expressed as mean ± SD and compared by ANOVA. For the TMA results, the data were entered into a computer database and the statistical analysis was performed using SPSS for Windows (version 22.0, IBM SPSS Inc., New York, NY). For continuous variables, analysis was done using Student's t-test and one-way ANOVA to determine if there was a significant difference in expression of the receptors between tissue groups. Fisher's exact test or likelihood ratio was used for analyzing the association between expression level of receptors and categorical clinicopathological variables. Survival analysis was performed by Kaplan-Meier method with the log-rank test. *P* values < 0.05 were considered as significant for all experiments.

## SUPPLEMENTARY MATERIALS AND METHODS


