# Supplementary material for: Targeting VEGFR1- and VEGFR2-expressing non-tumor cells is essential for esophageal cancer therapy
Source: Oncotarget. 2014 Dec 17;6(3):1790–805. doi: 10.18632/oncotarget.2781 (PMC4359332; doi:10.18632/oncotarget.2781)
Supplement: Supplementary file 1 [file oncotarget-06-1790-s001.pdf]

SUPPLEMENTARY MATERIALS AND METHODS

Cell proliferation assay

HUVECs (~ 5 × 10^3 cells) were seeded in 96-well plate and cultured with fresh M200 containing 0.4% (v/v) LSGS. Under the stimulation of recombinant human VEGF (20 ng/ml, Invitrogen), the cells were treated with IMC-18F1, IMC-1121B or a combination of both antibodies for 9 days during which the medium was refreshed every 3 days. After treatment, HUVECs were counted in 6 random fields under phase-contrast microscope. The proliferation of VEGF-stimulated mouse VEGFR1$^+$/VEGFR2$^+$ BMCs was determined in the same way after treatment with MF-1 or DC101. Proliferation of ESCC cells was determined using MTT assay [1] after treatment with anti-mouse VEGFR antibodies for 9 days or anti-human VEGFR antibodies for 3 days.

In vitro cell migration and invasion assay

The chemotactic mobility of HUVECs and sorted VEGFR1$^+$/VFGR2$^+$ BMCs was investigated using migration chamber assay as previously described [1]. Briefly, the cells were mixed with indicated concentrations of VEGFR1 and/or VEGFR2 neutralizing antibodies (IMC-18F1, IMC-1121B, MF-1 and DC101), then loaded into the upper compartment of the Boyden chamber (Millipore). VEGF (20 ng/ml) served as chemoattractant in the lower chamber. After 24 h, the migrated cells were fixed with methanol and stained with crystal violet, then quantified under the microscope in 6 random fields. BioCoat matrigel invasion chambers (BD Biosciences) were used to investigate the invasion of ESCC cells attracted by the conditioned media (CM) of VEGF-pretreated HUVECs or sorted VEGFR1$^+$ and VEGFR2$^+$ BMCs. Briefly, KYSE270 cells were loaded in the upper compartment while VEGF-stimulated VEGFR1$^+$ and VEGFR2$^+$ BMCs mixed with indicated concentrations of MF-1 and/or DC101 were seeded in the lower compartment. After 24 h, the invaded cells were fixed and stained as above, then quantified by submerging the chambers in 1% SDS and measuring the absorbance of the solution at 570 nm. Images of 3 random fields were captured from each membrane.

Endothelial tube formation assay

In brief, HUVECs mixed with desired concentrations of human monoclonal antibodies directed against human VEGFR1 (IMC-18F1) and/or human VEGFR2 (IMC-1121B) were seeded on matrigel in 96-well plate. After 6 hours, capillary-like tubes were imaged and the degree of tube formation was quantified by measuring the total tube length in 6 random fields from each well using the software Stereo Investigator (MBF Bioscience, Williston, VT).

Western blot analysis

The blots were probed with primary antibodies against: human/mouse phospho-VEGFR1 (Tyr1213) (#07-758; 1:100 dilution) purchased from Millipore (Billerica, MA); human VEGFR1 (#2893; 1:1000 dilution), human/mouse VEGFR2 (#2479; 1:1000 dilution), human/mouse phospho-VEGFR2 (Tyr1175) (#2478; 1:100 dilution), phospho-Erk (Thr202/Tyr204) (#4376; 1:1000 dilution), Erk (1:2000 dilution), Src (1:2000 dilution), phospho-EGF receptor (Tyr1068) (#2234; 1:1000 dilution), EGF receptor (#2646; 1:1000 dilution) and phospho-Src (Tyr416) (#6943; 1:1000 dilution) obtained from Cell Signaling Technology; human cytokeratin 8 (1:1000 dilution) from Epitomics (Burlingame, CA); mouse VEGFR1 (#MAB471; 1:1000 dilution) from R&D Systems; and actin (1:2000 dilution) from Santa Cruz Biotechnology.

In vivo matrigel plug assay

Briefly, 6–8 week old C57BL/6 mice were randomized into 7 groups (n = 3 per group). Growth factor reduced matrigel (BD Biosciences) mixed with heparin and different concentrations of MF-1 and/or DC101, with or without addition of VEGF, was subcutaneously injected into the flank of mice. After 7 days, the mice were sacrificed and the matrigel plugs were harvested for immunohistochemical analysis of MVD indicated by CD31 staining and hemoglobin evaluation using Drabkin's reagent kit (Sigma).

Preparation of cell suspensions from mouse bone marrow, tumor xenografts and lungs

Mouse BMCs were prepared according to Kuznetsov et al. [2]. In brief, the bone marrow of nude mice was collected by flushing the tibias and femurs with sterile DMEM/F-12 (Invitrogen). The mixture was passed through an 18-gauge needle and filtered through 70-μm nylon mesh cell strainer to dissociate the cells. Sorted VEGFR1$^+$/VEGFR2$^+$ BMCs were *ex vivo* expanded in DMEM/F-12. The tumor xenografts and lungs harvested from mice were minced to 1 mm^3 size and digested in collagenase (100 μg/ml) for 20 min at 37°C with continuous shaking.

Fluorescence-activated cell sorting and analysis

The stained samples were analyzed on a BD FACSCanto II Analyzer (BD Biosciences, San Jose, CA). The data analysis was carried out by using FlowJo software (Tree Star Inc., Ashland, OR). Bone marrow-derived VEGFR1^{+} cells and VEGFR2^{+} cells were sorted on a BD FACSAria I Cell Sorter (BD Biosciences). The top 20% of most brightly stained cells and the bottom 20% of most dimly stained cells were selected as positive and negative populations respectively.

Immunohistochemistry and evaluation of staining

Immunohistochemical analysis of proliferation index and micro-vessel density (MVD) was performed and evaluated as previously described [3]. In brief, paraffin-embedded sections of tumor xenografts collected from mice were deparaffinized and rehydrated in graded ethanol series. After incubation with 0.3% hydrogen peroxide for 30 min, antigen retrieval was performed by heating the slides in citrate buffer (pH 6.0) in a microwave oven. The sections were then blocked with normal serum, and incubated with primary antibodies against Ki-67 (Dako, Mississauga ON) and CD31 (Santa Cruz Biotechnology). Next, the slides were incubated with biotinylated secondary antibodies, followed by peroxidase-conjugated avidin-biotin complex. The immunoreactive signals were visualized using a 3, 3′-diaminobenzidine (Dako) substrate. Finally, the sections were counterstained with hematoxylin. The Ki-67 proliferation index was determined based on the mean percentage of positively stained nuclei in six randomly selected fields from representative tumor sections. MVD was calculated as the mean number of CD31-positive vessels stained in six random fields visualized under 20X objective.

SUPPLEMENTARY REFERENCES

1. Li B, Li YY, Tsao SW, Cheung AL. Targeting NF-kappaB signaling pathway suppresses tumor growth, angiogenesis, and metastasis of human esophageal cancer. Mol Cancer Ther. 2009; 8:2635–2644.
2. Kuznetsov HS, Marsh T, Markens BA, Castano Z, Greene-Colozzi A, Hay SA, et al. Identification of luminal breast cancers that establish a tumor-supportive macroenvironment defined by proangiogenic platelets and bone marrow-derived cells. Cancer Discov. 2012; 2:1150–1165.
3. Li B, Tsao SW, Li YY, Wang X, Ling MT, Wong YC, He QY, Cheung AL. Id-1 promotes tumorigenicity and metastasis of human esophageal cancer cells through activation of PI3K/AKT signaling pathway. Int J Cancer. 2009; 125:2576–2585.

SUPPLEMENTARY FIGURES

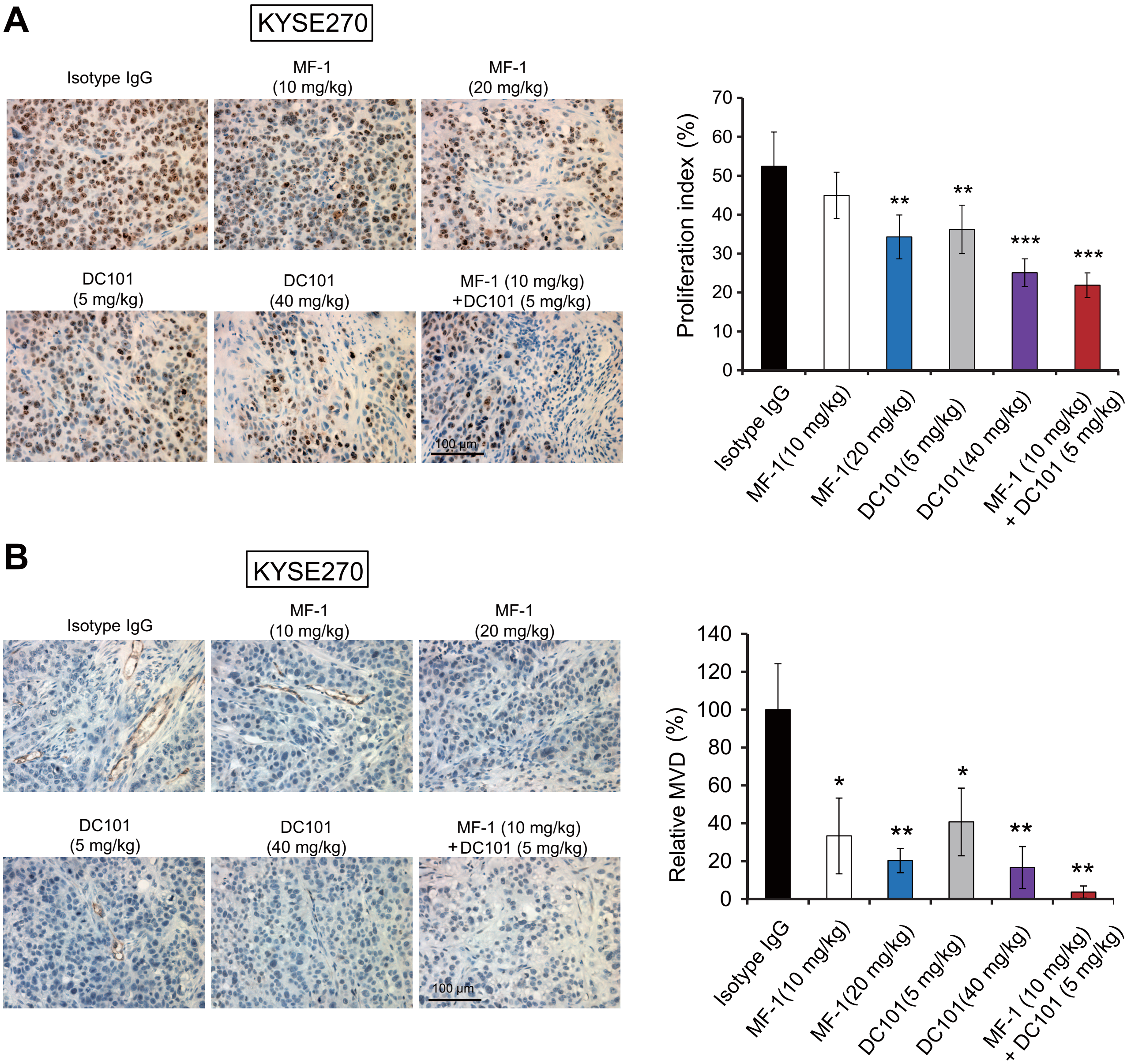

Supplementary Figure S1: Immunohistochemical analysis of Ki-67 and CD31 in KYSE270 tumor xenografts. (A, B) Determination of Ki-67 proliferation index (A) and CD31-positive MVD (B) in the KYSE270 tumor xenografts. Bars, SD; *, $P < 0.05$; **, $P < 0.01$, ***, $P < 0.001$, compared with isotype IgG-treated mice.

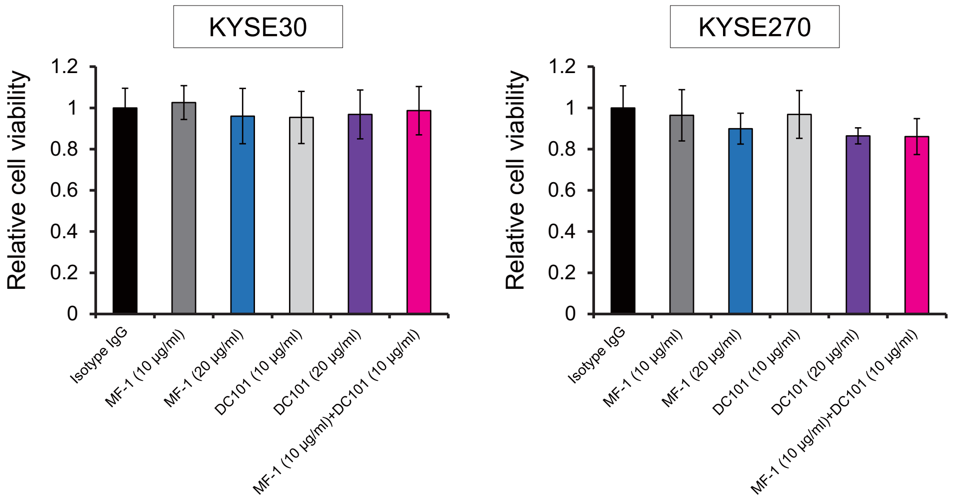

Supplementary Figure S2: MF-1 and DC101 had no significant effect on proliferation of human ESCC cells *in vitro*.

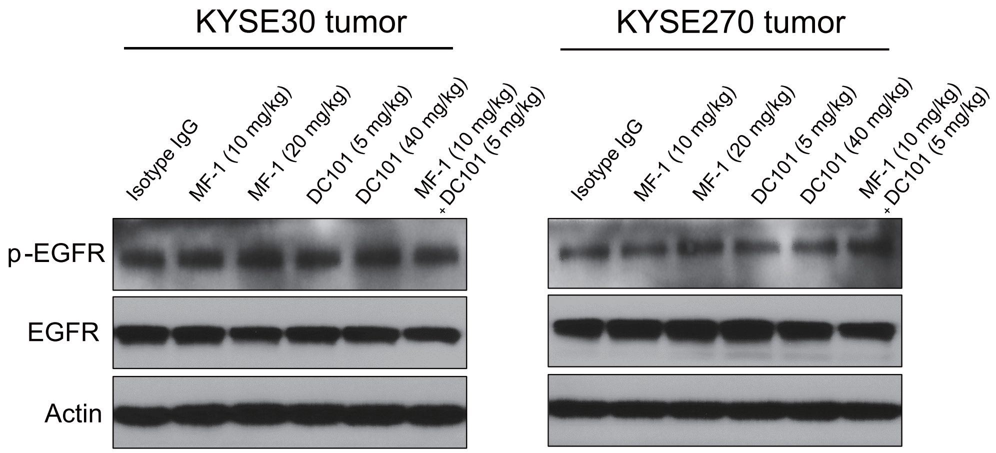

Supplementary Figure S3: Western blot analysis of the expression levels of p-EGFR (Tyr1068) and EGFR in the KYSE30 and KYSE270 tumor xenografts.

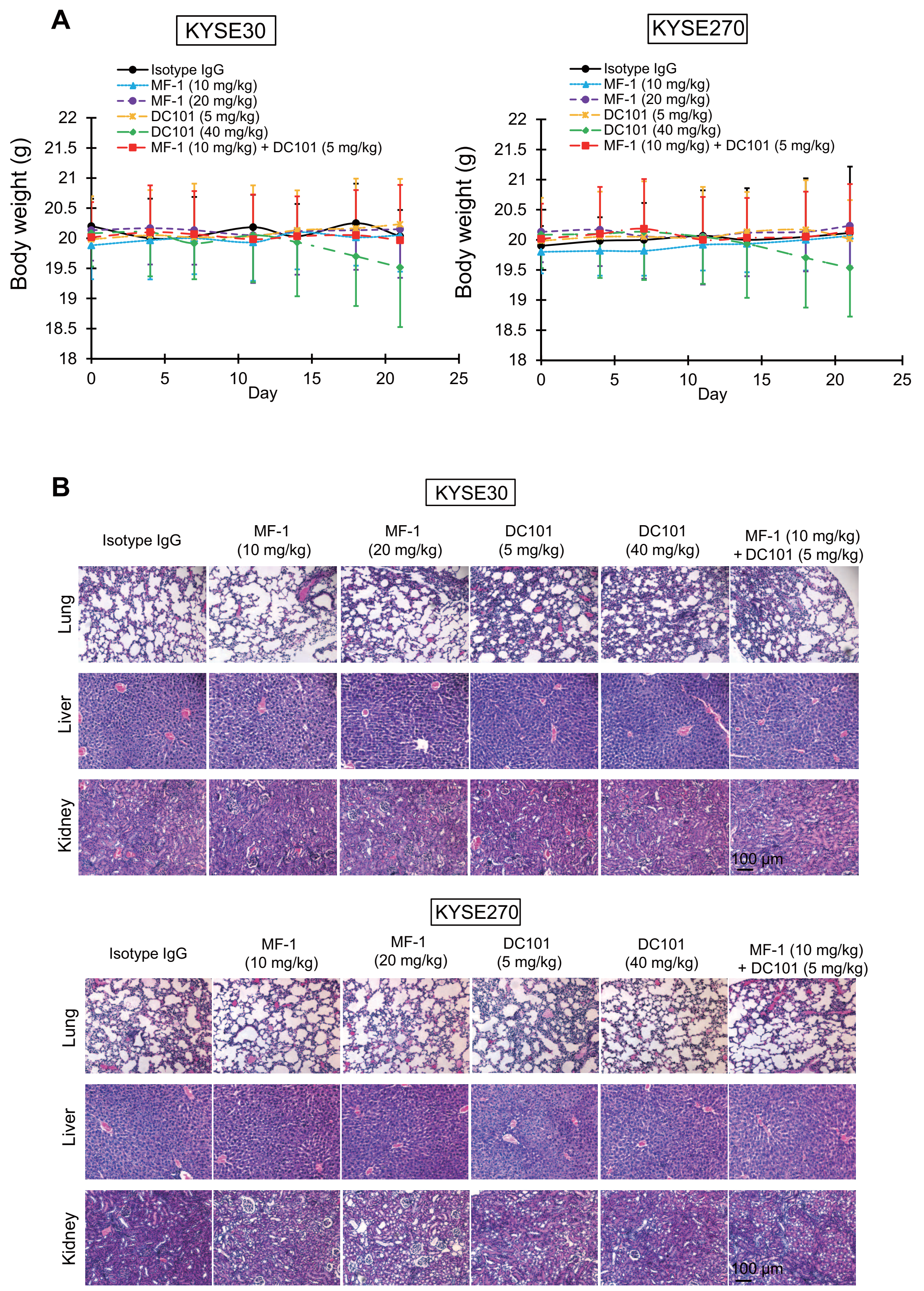

Supplementary Figure S4: Evaluation of side effects of VEGFR1 and VEGFR2 antibodies in nude mice. **(A)** Body weight of mice during the experimental period. Bars, SD. **(B)** Representative hematoxylin and eosin stained sections of lung, liver and kidney.

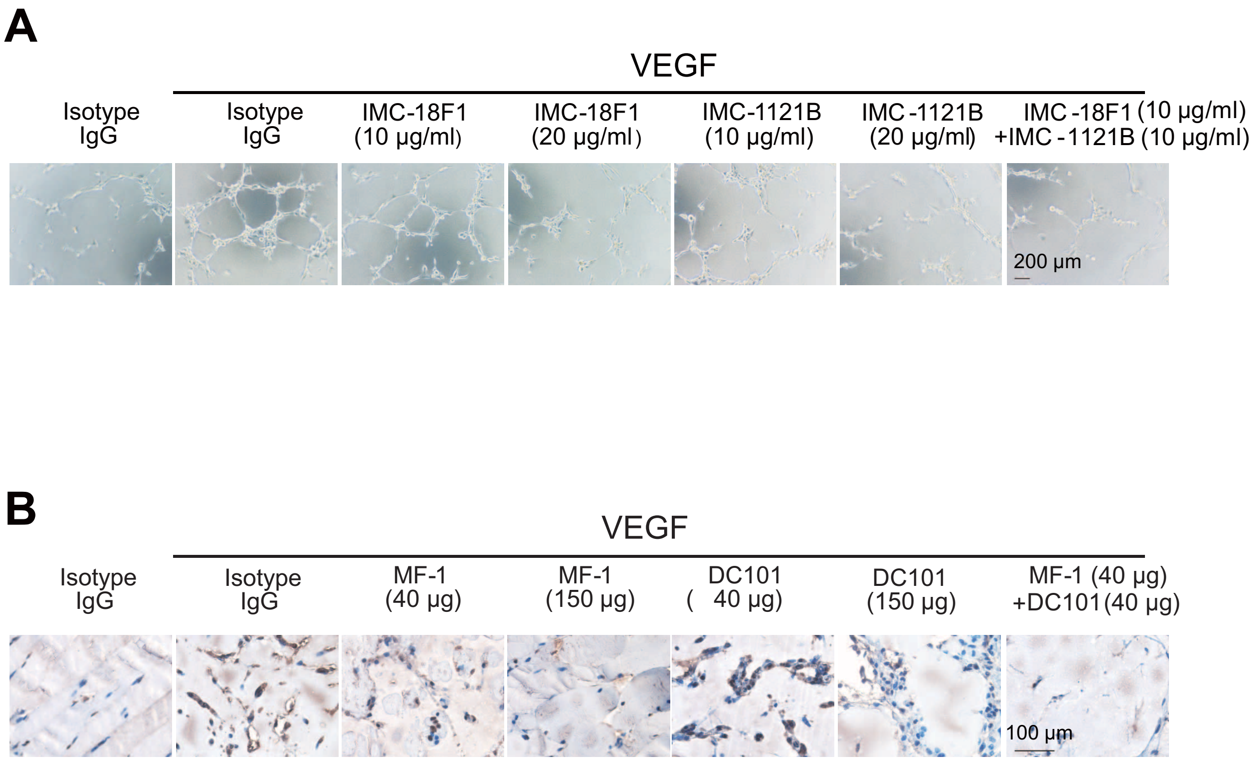

Supplementary Figure S5: (A) Representative images of HUVEC tube formation assay shown in Figure 2C. **(B)** Representative images of CD31-immunostained micro-vessels corresponding to MVD shown in Figure 2F.

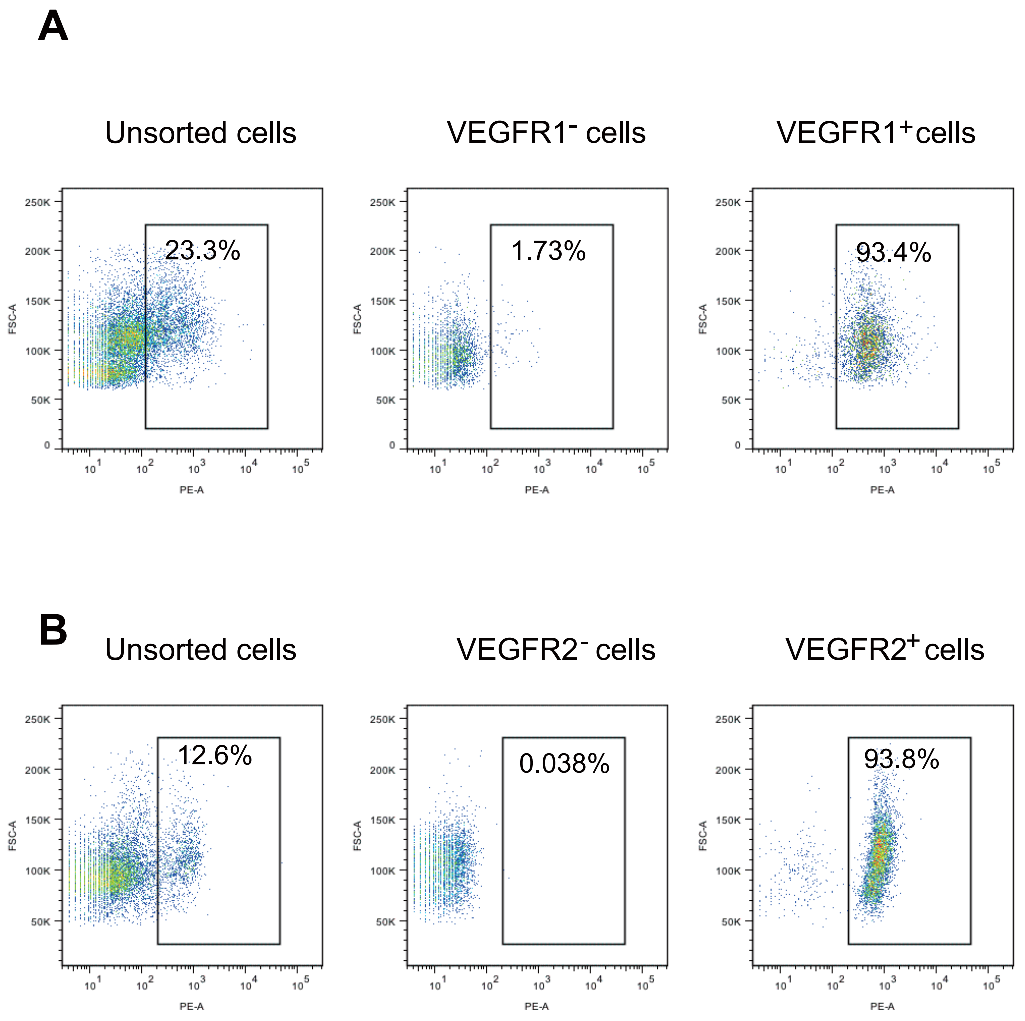

Supplementary Figure S6: Post-sort analysis of VEGFR1^{+} and VEGFR2^{+} BMCs.
